# Long-Term Predictive and Feedback Encoding of Motor Signals in the Simple Spike Discharge of Purkinje Cells

**DOI:** 10.1523/ENEURO.0036-17.2017

**Published:** 2017-04-11

**Authors:** Laurentiu S. Popa, Martha L. Streng, Timothy J. Ebner

**Affiliations:** Department of Neuroscience, University of Minnesota, Minneapolis, MN 55455

**Keywords:** cerebellum, forward internal model, kinematics, motor errors, Purkinje cell, working memory

## Abstract

Most hypotheses of cerebellar function emphasize a role in real-time control of movements. However, the cerebellum’s use of current information to adjust future movements and its involvement in sequencing, working memory, and attention argues for predicting and maintaining information over extended time windows. The present study examines the time course of Purkinje cell discharge modulation in the monkey (*Macaca mulatta*) during manual, pseudo-random tracking. Analysis of the simple spike firing from 183 Purkinje cells during tracking reveals modulation up to 2 s before and after kinematics and position error. Modulation significance was assessed against trial shuffled firing, which decoupled simple spike activity from behavior and abolished long-range encoding while preserving data statistics. Position, velocity, and position errors have the most frequent and strongest long-range feedforward and feedback modulations, with less common, weaker long-term correlations for speed and radial error. Position, velocity, and position errors can be decoded from the population simple spike firing with considerable accuracy for even the longest predictive (-2000 to -1500 ms) and feedback (1500 to 2000 ms) epochs. Separate analysis of the simple spike firing in the initial hold period preceding tracking shows similar long-range feedforward encoding of the upcoming movement and in the final hold period feedback encoding of the just completed movement, respectively. Complex spike analysis reveals little long-term modulation with behavior. We conclude that Purkinje cell simple spike discharge includes short- and long-range representations of both upcoming and preceding behavior that could underlie cerebellar involvement in error correction, working memory, and sequencing.

## Significance Statement

Most hypotheses of cerebellar function emphasize a role in real-time control of movements. However, contributions to adjustment of future movements, sequencing, working memory, and attention argue for the cerebellum's ability to predict and maintain information over an extended time frame. This is the first single unit recording study showing that the simple spike discharge of Purkinje cells contains long-range representations of motor parameters. These signals may provide the neural substrate underlying the cerebellar contributions to movement control over an extended temporal horizon and a potential mechanism unifying aspects of cerebellar function in motor and nonmotor domains.

## Introduction

The cerebellum plays a major role in the control of precise and skillful motor behaviors. The time frame for expression of cerebellar function in motor control is generally assumed to be relatively brief, centered around current movement (Braitenberg, 1967; Eccles et al., 1967; Brooks and Thach, 1981; Welsh et al., 1995; Ivry and Spencer, 2004; Llinas, 2013). However, several observations suggest that the cerebellum contributes to behavior over longer time periods. The CNS uses information about a current movement, including errors, to adjust subsequent behavior and the cerebellum has a central role in this process for arm and eye movements (Miall and Wolpert, 1996; Diedrichsen et al., 2005; Tseng et al., 2007; Shadmehr et al., 2010; Taylor et al., 2010; Popa et al., 2012; Schlerf et al., 2012). The use of past performance information involves multiple time scales, including a relatively short time course process that responds well to error but has poor retention and a longer process that responds weakly to error but retains the information well (Smith et al., 2006; Yang and Lisberger, 2010). In the faster process, errors are represented as a time decaying “trace” with a time constant of ∼4 s for reaching movements (Huang and Shadmehr, 2007), ∼4-10 s for smooth pursuit (Yang and Lisberger, 2010), and ∼15 s for saccades (Chen-Harris et al., 2008). During reaching movements, the feedback response to an error appears to serve as a template to adapt the subsequent motor command (Albert and Shadmehr, 2016). Therefore, motor error and/or performance information must be retained over many seconds.

The cerebellum is a candidate for this short-term memory of motor performance (Smith and Shadmehr, 2005; Huang and Shadmehr, 2007; Yang and Lisberger, 2010). One possibility is that the memory utilizes one of the many forms of synaptic plasticity found in cerebellar circuits (for reviews, see Hansel et al., 2001; Ito, 2001; Boyden et al., 2004; Gao et al., 2012). In addition to synaptic plasticity, another possibility is that persistent activity in cerebellar neurons provides a type of working memory, as commonly observed in the cerebral cortex (for review, see Mongillo et al., 2008; Gazzaley and Nobre, 2012; Nyberg and Eriksson, 2015; D’Esposito and Postle, 2015), which conveys information about past performance to inform upcoming movements.

Imaging and lesion studies implicate the cerebellum in sequencing and working memory tasks in both the motor and cognitive domains (Doyon et al., 1997; Molinari and Petrosini, 1997; Molinari et al., 1997; Lu et al., 1998; Nixon and Passingham, 2000; Leggio et al., 2008; Molinari et al., 2008). Sequencing requires planning actions in advance as well as monitoring and storing past actions over several seconds (Haggard, 1998; Desrochers et al., 2015). Cerebellar activation occurs in working memory tasks that require maintaining and recalling motoric and/or nonmotoric information over long-range time scales (Chen and Desmond, 2005; Hautzel et al., 2009; Marvel and Desmond, 2010; Küper et al., 2016). Also, cerebellar activation precedes movement, sensory stimuli, and cognitive tasks by several seconds (Tesche and Karhu, 2000; Hülsmann et al., 2003; Ghajar and Ivry, 2009). Therefore, we hypothesize that cerebellar neurons may convey feedforward and feedback signals of behavior over a time span of seconds.

Historically, studies of the motor information conveyed by cerebellar neurons during arm and eye movements have focused on short time frames (Marple-Horvat and Stein, 1987; Fortier et al., 1989; Stone and Lisberger, 1990; Shidara et al., 1993; Fu et al., 1997; Gomi et al., 1998; Coltz et al., 1999; Roitman et al., 2005; Medina and Lisberger, 2009; Dash et al., 2012). Only a few single neuron studies have examined longer periods linked to planning, working memory, or error processing. Dentate neurons show significant premovement activity over several hundred milliseconds during instructed saccade and reach sequencing tasks (Mushiake and Strick, 1993; Middleton and Strick, 1998; Ashmore and Sommer, 2013; Kunimatsu et al., 2016); however, these investigations did not evaluate longer time epochs. Beyond the observed differences in pro- versus antisaccades before movement (Kunimatsu et al., 2016), it is not clear to what degree this premovement activity encodes upcoming kinematics.

Most experimental paradigms used to examine the time course of cerebellar neuronal modulation have relied on brief, discrete, and stereotypical movements that likely do not require extended planning or monitoring of past actions. In contrast, here, we use a pseudo-random tracking task that involves continuous movements and requires constant error processing and corrections, allowing investigation over several seconds of the motor representations previously described in Purkinje cell simple spike discharge (Hewitt et al., 2011; Popa et al., 2012).

## Materials and Methods

All animal experimentation was approved by the Institutional Animal Care and Use Committee at the University of Minnesota and conducted in accordance with the guidelines of the National Institutes of Health. Data were collected using the same pseudo-random tracking paradigm, animal preparation, and recording procedures described in two previous papers (Hewitt et al., 2011; Popa et al., 2012). Two head-fixed Rhesus monkeys (one male: monkey I, one female: monkey N) used a robotic manipulandum (InMotion^2^) to control a cross-shaped cursor to track a circular target (2.5 cm in diameter) on a computer screen mounted at eye level 50 cm in front of the animal. A set of 100 pseudo-random target paths were generated from a smoothed sum of sine waves, under the constraint that the target’s random speed followed the two-thirds power law (Viviani and Terzuolo, 1982; Lacquaniti et al., 1983). The duration of the track period ranged from 6 to 10 s. In addition, each trajectory had a different start and end position, with a required hold time at the start (initial hold) and end positions (final hold) that ranged from 1000-3000 ms. The paradigm required that the animal maintain the cursor within the target and allowed for only brief excursions outside the target (<500 ms). During each recording session, the presentation order of the trajectories was randomized. The animal’s view of their arm was blocked and performing the task depended on visually monitoring the target and cursor movements on the screen.

Cursor/hand and target center positions were sampled and recorded at 200 Hz. Error and kinematic parameters (see below) were derived from the hand position (down sampled to 50 Hz and filtered using a second order Butterworth filter with a 3-Hz cutoff). Full head MRI and CT images in Monkey Cicerone were used to model recording chamber locations and electrode penetrations (Miocinovic et al., 2007). Models for both monkeys showed electrode recording positions mostly in lobules IV–V of the intermediate zone and neighboring lateral zones, anterior to the primary fissure. Arm and hand movement-related activity has been well established in the recorded region (Thach, 1968; Mano and Yamamoto, 1980; Fortier et al., 1989; Fu et al., 1997; Roitman et al., 2005; Pasalar et al., 2006; Yamamoto et al., 2007; Hewitt et al., 2011).

After full recovery from chamber implantation surgery, extracellular recordings were obtained using Pt-Ir electrodes with parylene C insulation (0.8-1.5 MΩ impedance, Alpha Omega Engineering) that were inserted just deep enough to penetrate the parietal dura using a 22-Gauge guide tube. Electrodes were advanced to the cerebellum using a hydraulic microdrive (Narishige Group). Purkinje cells were identified by the presence of both simple spike and complex spike discharge, with the characteristic brief simple spike inactivation following a complex spike. Electrophysiological data were sampled at 32 kHz. Simple spikes were discriminated online using the Multiple Spike Detector System (Alpha Omega Engineering) after conventional amplification and filtering (30-Hz to 3-kHz band pass, 60-Hz notch). Discriminated simple spike trains were transformed into instantaneous firing rate at 50-Hz sampling rate, and low-passed filtered also using a second order Butterworth filter with a 3-Hz cutoff. Finally, the mean simple spike firing rate was subtracted.

**Figure 1.**
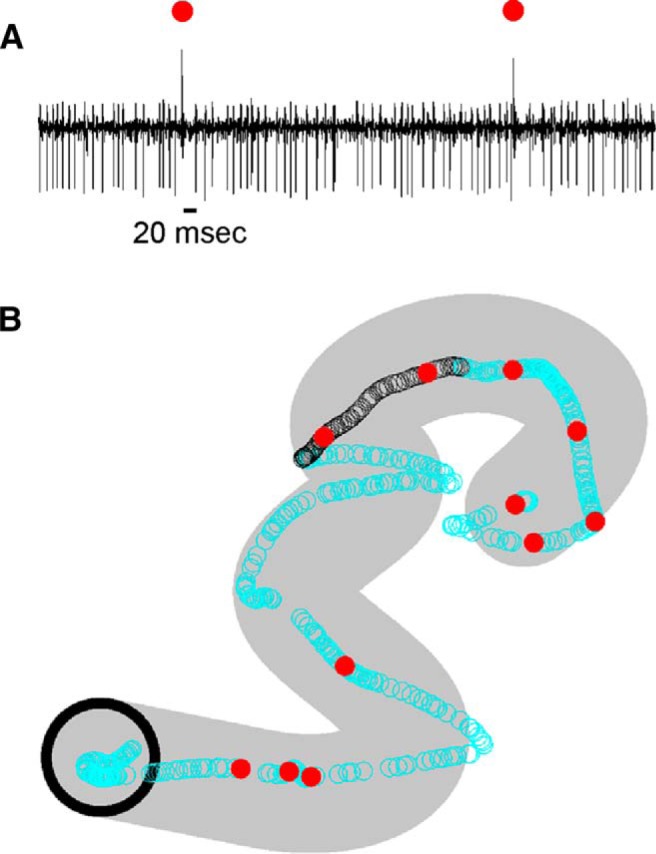
Example of a Purkinje cell recording during pseudo-random tracking. ***A***, One-second Purkinje cell recording showing both **s**imple spikes and complex spikes (marked by red dots). ***B***, Occurrence times of simple spikes (open circles) and complex spikes (red dots) superimposed on the hand position for one trial. Black open circles correspond to the simple spikes from the 1-s data segment shown in ***A***. Initial target position marked by the black circle (target diameter, 2.5 cm). Area covered by target movement during the trial shown in gray.

As in previous studies (Hewitt et al., 2015; Streng et al., 2017), complex spikes were manually identified and discriminated off-line from the raw electrophysiological recordings by the presence of the characteristic wave form including an initial large amplitude spike followed by spikelets (see Figure 1*A*). Complex spike identification also required the characteristic pause in simple spike firing (Thach, 1967; Bloedel and Roberts, 1971). A custom MATLAB graphical user interface recorded the time stamp of the initial spike (1-ms resolution). For quantitative analysis of the complex spike modulation with behavior, a subset of 40 Purkinje cells was selected based on consistent isolation and discrimination of the complex spike discharge throughout the entire recording session.

### Kinematics and performance errors during pseudo-random tracking

Arm movements were described by five kinematic parameters: position (X, Y), velocity (VX, VY), and speed (S) based on the trajectory of a cursor mapping the arm movement in the horizontal plane on a vertical screen. The velocity vector [VX(t), VY(t)] components were computed by numerical differentiation of the corresponding position data. Speed [S(t)] was computed as the magnitude of the velocity vector. Position errors [XE(t), YE(t)] were defined as the difference between the cursor and center of the target, and radial error [RE(t)] defined as the magnitude of the position error vector. As shown previously, the animals use these measures to perform the task as they strive to minimize these errors (Popa et al., 2012). We did not include either velocity or speed errors as these parameters were highly correlated with velocity and speed as discussed previously for this tracking task (Popa et al., 2012).

### Linear modeling of simple spike firing based on firing residuals during tracking

The aim of this study was to determine whether the simple spike discharge encodes kinematic or error parameters over extended time periods. Linear modeling was used to relate the simple spike firing with the kinematic and error parameters, as previously used in the same task (Popa et al., 2012) and during reaching movements (Hewitt et al., 2015). For the first analysis, linear regressions were performed using only the firing during the actual tracking and did not include the firing in the hold periods. To evaluate the individual parameters in isolation, firing residuals were obtained from a multilinear model that included all kinematic or error terms not being evaluated. The purpose of this regression is to remove the variability in the firing related to the kinematic and error parameters other than the parameter of interest. For example, to evaluate VX, we regressed the firing to a multilinear model that included all other parameters (X, Y, VY, S, XE, YE, and RE) as independent variables. The resultant firing residuals from this regression are independent of the model parameters. Therefore, regressing the firing residuals with VX is not confounded by the covariance between VX and the rest of the parameters. All regressions used to obtain firing residuals were computed using the instantaneous firing and movement data from individual trials (i.e., nonaveraged data). Firing residuals were determined at each of the 20-ms time shifts (τ) between the simple spike firing and the model predictors in a -2000 to 2000-ms window. This process was repeated for each kinematic and error parameter, first computing the firing residuals of interest followed by fitting the firing residuals to the chosen motor parameter.

For this regression analysis, we used an averaging method similar to the previous characterization of the simple spike modulation with kinematics and errors. The partitions of the kinematic and error workspaces were chosen to result in approximately a similar number of bins to minimize the variability in the regression analyses, as the coefficient of variation (R^2^) is highly dependent on the number of bins (Freund, 1971; Zar, 1999). The kinematic space (X, Y, VX, VY, S) was partitioned in five equal bins along each dimension (2.4-cm bins from -6 to 6 cm for X and Y, 4.8-cm/s bins from -12 to 12 cm/s for VX and VY, and 2.4-cm bins from 0 to 12 cm/s for S) resulting in 3125 five-dimensional bins. Error parameters were partitioned into 16 equal bins along each dimension (0.375-cm bins from -3 to 3 cm for XE, YE, and 0.2-cm bins from 0 to 3.2 cm for RE) resulting in 4096 three-dimensional bins. The neural data were sorted and averaged into these kinematic and error partitions. To be included in further analyses, a bin had to contain >20 observations.

All linear model analyses used repeated fittings in which the dependent data series (firing residuals or actual firing) were shifted relative to the model’s independent variable(s) data series (Ashe and Georgopoulos, 1994; Gomi et al., 1998; Paninski et al., 2004; Medina and Lisberger, 2009; Roitman et al., 2009; Popa et al., 2013). The temporal shifts were used to assess the lead/lag (τ) between the neural modulation (i.e., neural signals) and behavioral parameters (i.e., errors or kinematics). We used a much longer time window (-2000-2000 ms) than has typically been used in these temporal analyses. Note that the longer window requires reducing the length of the data series, which depends on trial duration. The regression analyses resulted in R^2^ and β profiles in time for each kinematic and error parameter. The β values provide a measure of the firing sensitivity with the parameters. Modulation with a motor parameter was defined by the presence of a significant local maxima in the R^2^ profile. At each maxima, the R^2^ value quantifies the strength of encoding, the corresponding β value quantifies the signal sensitivity and the τ value determines the signal timing relative to behavior. Negative τ values indicate that the neural modulation led the model regressors.

We used a bootstrap approach to define significance of the regression results. For each Purkinje cell recording, the trial order was shuffled so that the simple spike firing was uncoupled from the behavior. The shuffling followed by the entire regression analysis was repeated 100 times for each recording session, and the mean and SD of the R^2^ profile from the shuffled regressions determined for each τ value. To be considered statistically significant, the R^2^ local maxima from the unshuffled firing had to exceed the mean + 4 SD of the shuffled trials.

The bootstrapped R^2^ distribution is not normal, as determined using a Kolmogorov–Smirnov test. Therefore, we determined the probability that the bootstrapped R^2^ distribution exceeds the significance threshold of 4 SD, which was equivalent to an estimated *p* value of 0.02. This approach minimizes the problem of spurious correlation due to autocorrelation in the firing and behavioral parameters (Granger and Newbold, 1974; Tagaris et al., 1997; Leuthold et al., 2005; Christova et al., 2011; Lewis et al., 2012). The -2000- to 2000-ms window was divided into eight 500-ms epochs including four predictive epochs: -2000 to -1500 (P1), -1500 to -1000 (P2), -1000 to -500 (P3) and -500-0 ms (P4) and four feedback epochs: 0-500 (F1), 500-1000 (F2), 1000-1500 (F3), and 1500-2000 ms (F4). Within each epoch, the occurrence and magnitude of significant simple spike modulation with the behavioral parameters were determined.

### Linear modeling of simple spike firing based during the hold periods

We also investigated the behavioral representations in the simple spike activity during the initial and final hold periods that preceded and followed the track period of each trial, respectively.

The analyses followed the overall approach used for the track period to linearly model the simple spike firing with kinematics and errors. The goals were to determine whether the simple spike firing during the initial hold encoded predictive information about the upcoming track period and whether the simple spike firing during the final hold encoded feedback information about the end of the track period. Firing during the initial hold period was regressed to the kinematic and error parameters defined above (X, Y, VX, VY, S, XE, YE, RE) in a sliding window of the same duration as the initial hold period. This behavioral window was moved in 20-ms steps (τ value) from 0 ms (i.e., window overlaps entirely with the initial hold period) to 2000 ms in the upcoming tracking period. Using the same trial shuffled bootstrap approach and significance criteria as in the track period, significance was defined as the R^2^ exceeding the mean + 4 SD of the shuffled trials. Note that for consistency with the track period analysis, we used the same temporal notation in which negative τ values denote predictive encoding. For summary and the decoding analyses, the results of regressions results from the simple spike firing in the initial hold period were divided into four predictive epochs: -2000 to -1500 (P1), -1500 to -1000 (P2), -1000 to -500 (P3), and -500-0 ms (P4). Similarly, we regressed the simple spike firing during the final hold period against each parameter using a sliding window of the same duration as the final hold period. The behavioral window was moved in 20-ms steps from 0 ms (i.e., overlapping entirely with the holding period) to 2000 ms into the preceding track period (Fig. 10*C*). Positive τ values were used to denote feedback encoding. For summary and the decoding analyses, the regressions results from the simple spike firing in the final hold period were divided into four feedback epochs: 0-500 (F1), 500-1000 (F2), 1000-1500 (F3), and 1500-2000 ms (F4).

### Display of simple spike firing

**Figure 2.**
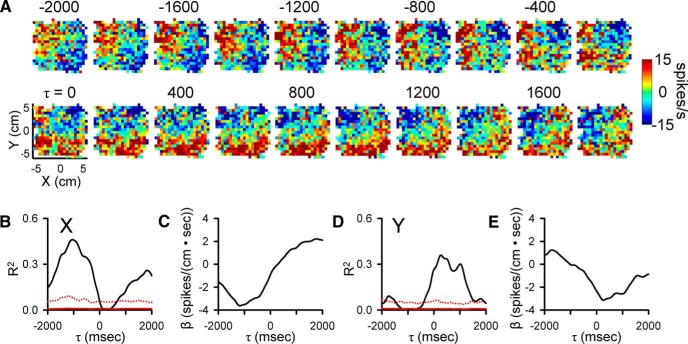
Purkinje cell simple spike modulation in relation to hand position during the track period. ***A***, Sequence of firing maps in 200-ms steps showing simple spike modulation with hand position. Map colors denote simple spike rate relative to mean firing rate, according to color bar. ***B***, ***D***, R^2^ temporal profiles show the strength of X and Y encoding as function of τ value. Chance level of simple spike encoding determined by trial shuffling, mean (red continuous line) + 4 SD (red dotted line). ***C***, ***E***, β temporal profiles show the simple spike sensitivity to X and Y as a function of τ value. In all figures, negative τ values represent firing leading behavior.

**Figure 3.**
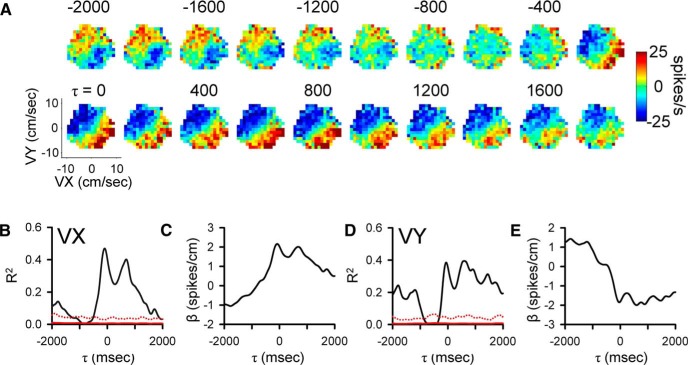
Purkinje cell simple spike modulation in relationship to hand velocity during track period. ***A***, Sequence of firing maps showing simple spike modulation with velocity in 200-ms steps. ***B***, ***D***, R^2^ temporal profile shows the strength of VX and VY encoding as function of time, respectively. ***C***, ***E***, β temporal profiles show the firing sensitivity to VX and VY as a function of τ, respectively. Color scheme of firing maps, τ values, and denotation of chance encoding as in Figure 2.

**Figure 4.**
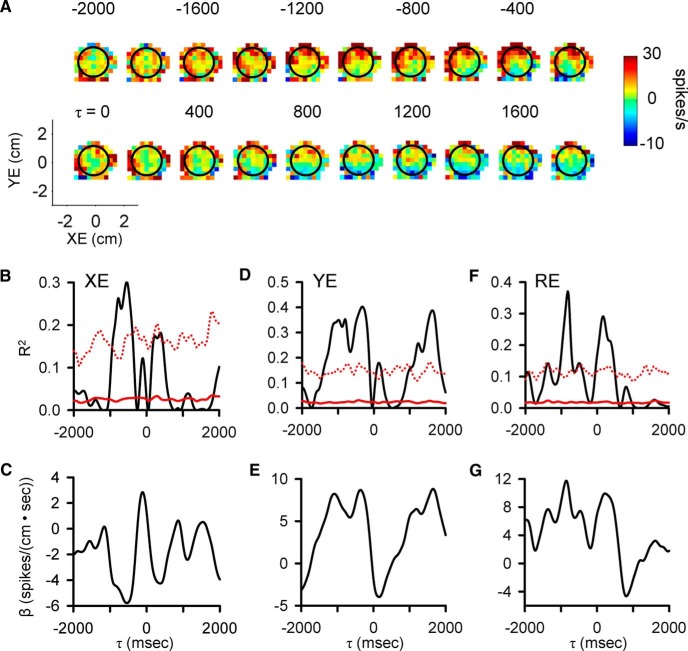
Purkinje cell simple spike modulation in relationship to position error during track period. ***A***, Sequence of firing maps showing simple spike modulation with position errors in 200-ms steps. ***B***, ***D***, ***F***, R^2^ temporal profile shows the strength of XE, YE, and RE encoding as function of τ, respectively. ***C***, ***E***, ***G***, β temporal profiles show the firing sensitivity to XE, YE, and RE as a function of τ, respectively. Color scheme of firing maps, τ values, and denotation of chance encoding as in Figure 2.

To display simple spike firing during the track period in relation to the parameters (see Figures 2–4), we generated two-dimensional maps at the different τ values. These track period firing maps divided the parameter workspaces into 20 equal bins along both dimensions (0.6-cm bins from -6 to 6 cm for position, 1.2 cm/s bins from -12 to 12 cm/s for velocity, and 0.3 cm from -3 to 3 cm for position errors). Similarly, to display simple spike firing during the hold periods two dimensional maps were generated at different τ values. Because the hold periods are considerably shorter in duration than the track periods, they contain substantially fewer data points. Therefore, the firing maps were divided into only 10 equal bins along both dimensions: 1.2-cm bins from -6 to 6 cm for position, 2.4-cm/s bins from -12 to 12 cm/s for velocity, and 0.5 cm from -2 to 2 cm for position errors. Note that the binning used for display of the simple spike firing for both track and hold periods is at a different resolution than that used for the linear regression modeling described above. This difference in partitioning reflects the challenges posed in analyzing a high dimensional behavior in which the five kinematic parameters and three error parameters were analyzed as two groups. Therefore, the regression analyses had to use higher dimensional partitions that somewhat limited resolution. Conversely, the firing plots are based on two-dimensional maps (e.g., X and Y, VX and VY, and XE and YE) that allowed higher resolution partitioning.

### Control analyses

An important issue is whether the behavior or simple spike firing have an inherent temporal structure that can account for any significant correlations. Although the use of residuals ensures that other parameters are not influencing the firing at each τ value, there remains the possibility that the individual time series have inherent intertemporal correlations. To address this potential confound, several analyses were performed. First, the autocorrelation of each behavioral parameter and the simple spike firing was determined with the ±2000-ms window for each trial and then averaged across all recording sessions to evaluate the presence of local minima or maxima. Second, cross-correlations between all pairs of behavioral parameters were calculated within the ±2000-ms window to assess for lagged relationships.

Third, we reasoned that if the behavioral interactions between a pair of parameters determine the simple spike encoding then the β profiles for the parameters will be similar to the behavioral interaction and the degree of similarity will vary with the strength of the behavioral correlation. Therefore, for pairs of behavior with positive or negative cross-correlation values exceeding a threshold (*ρ* < -0.1 or *ρ* > 0.1), we determined the degree to which the simple spike encoding depended on the behavioral correlation. We quantified the “encoding similarity” of the β profiles by computing their cross-correlation within the ±2000-ms interval. The peak of the positive behavioral interaction exceeding the threshold (*ρ* > 0.1) was correlated with the maximum simple spike encoding similarity for all 183 Purkinje cells. The minimum of any negative behavioral interaction exceeding the threshold (*ρ* < -0.1) was correlated with the minimum simple spike encoding similarity across the population.

### Decoding of motor parameters in different time windows

To determine the accuracy of the information encoded in the simple spike firing, we performed a linear decoding analysis. For the firing during the track periods, the decoding determined the ability of the simple spike firing across the population to reconstruct either the upcoming or past behavior. For the firing during the initial hold period, the decoding determined the ability to reconstruct the upcoming behavior and for the final hold period the past behavior. To faithfully maintain the normal physiology, we decoded the actual firing and not the residuals used in the linear regression analysis described above. For each cell, the trials were randomly divided into training (80%) versus test (20%) trials. The temporal linear regression analyses were repeated for each individual parameter using only the training trials. Statistical significance was determined using the bootstrap method described above. The decoding analysis used only the largest significant R^2^ peak in each of the 500-ms predictive and feedback epochs. For each peak R^2^, we inverted the regression equation using the regression coefficient (β) at the corresponding τ value to calculate the predicted behavior from the simple spike firing. For each behavioral parameter and time epoch, the decoded behavior was weighted by the ratio between the peak R^2^ relative to the mean of all R^2^ peaks in the epoch of interest across all neurons. For each parameter and epoch, the observed and decoded values were pooled across the cell population and sorted by the observed values into 20 equal bins spanning the range of the parameter of interest. Bins were averaged to obtain both the decoded estimate and observed values of the kinematic and performance errors. For each parameter this process was repeated 25 times and for each repeat the training and the test trial sets were randomly selected. Due to the low number of data points around the boundaries of the parameter workspace, we restricted the decoding to ±5.6 cm for X and Y, ±10 cm/s for VX and VY, ±2 cm for XE and YE, 0-2.2 cm for RE, and 0.4-9 cm/s for S.

Two measures were used to quantify the accuracy of decoding. The first, goodness of fit (GoF), was determined over the 25 decoding repeats:

$$GoF=1-\frac{\sum {\left(X_{Dec}-X_{Obs}\right)}^{2}}{\sum {\left(X_{Obs}-{\bar{X}}_{Obs}\right)}^{2}}$$

in which $X_{Dec}$ and $X_{Obs}$ are the decoded and the observed values, respectively, and ${\bar{X}}_{Obs}$ is the mean of the observed values (Best et al., 2016). Similar to the R^2^, GoF quantifies the variability common to both the observed and decoded values. A GoF of 1 denotes perfect decoding, while negative values denote inconsistent decoding, in which the variability of the decoded distribution greatly exceeds the variability of the observed. The second measure was the slope of the decoded values with the observed values across the 25 repeats as determined by a linear regression. A slope of 1 denotes perfect decoding. As an additional control, we assessed decoding performance against random decoding. For random decoding, instead of using the regression model determined by the training trials from a given cell, we used a model from a random cell and then followed the same procedures to estimate the decoding quality.

### Relationship between complex spike discharge and behavior

The low frequency of complex spike discharge is not compatible with the temporal linear regression analyses used for the simple spike firing in this report. Therefore, investigation of the relationship between complex spike discharge and each parameter was based on the complex spike triggered averaging strategy used recently (Streng et al., 2017). For each behavioral parameter, the average behavior was determined from 2000 ms before to 2000 ms after each complex spike, the same time window used for the simple spike analyses.

To test for a statistically significant relationship between the complex spike discharge and a parameter, a bootstrapping approach was used in which a noise distribution of the behavior was generated by randomly shuffling the interspike intervals of the complex spikes in each trial (ISI-shuffled, 50 repeats). Next, we computed the mean and standard deviation of the ISI-shuffled complex spike-triggered behavior. If the local minima or maxima of the complex spike-triggered averaged behavior exceeded a threshold of mean ± 4 SD of the noise distribution, the complex spike modulation with that parameter was defined as statistically significant. Note that, to be consistent with the threshold used for simple spike long-term correlations, the threshold (mean ± 4 SD) for complex spike modulation employed here is higher than that used in the previous study (Streng et al., 2017). Both the occurrence of a significant complex spike-coupled behavioral change and timing of the change were determined, with negative times indicating a behavioral change before and positive times indicating behavioral change after complex spike discharge.

## Results

### Long-term simple spike modulation with kinematics and performance errors during tracking

We recorded and analyzed the simple spike activity from 183 Purkinje cells in two monkeys (65 neurons in monkey N and 118 in monkey I) during the pseudo-random tracking task. This dataset includes a reanalysis of 120 neurons used to describe the kinematic and error signals in Purkinje cells (Hewitt et al., 2011; Popa et al., 2012). The neurons were recorded in lobules IV–VI of the paravermal and neighboring lateral cerebellar zones. An example Purkinje cell recording of simple spikes and complex spikes (red dots) during tracking is illustrated in Figure 1*A*, in which the 1-s sample period includes two complex spikes both followed by a simple spike inactivation period (Thach, 1967; Bloedel and Roberts, 1971). For the same cell in Figure 1*B*, the simple spikes and complex spikes during an entire trial are superimposed on the hand position (open circles and dots, respectively) with the area covered by movement of the target also shown (gray region). The initial analysis was restricted to the simple spike firing and behavioral parameters during the track period. The analysis not only confirmed the previously detailed short-term correlations in the simple spike firing at leads and lags within ±500 ms (Hewitt et al., 2011; Popa et al., 2012), but also revealed correlations at much longer times for both kinematics and errors. As illustrated for an example Purkinje cell (Fig. 2*A*), the plots of mean subtracted simple spike firing show modulation with position (X and Y) throughout the ±2000-ms window. As early as -2000 ms, the simple spike firing leads position, with the strongest modulation in the upper left quadrant that peaks about -1500 ms. The firing pattern evolves from feedforward to feedback lags, with the predominant modulation in the lower half of the position space from 0-1000 ms. The R^2^ and β temporal profiles for X (Fig. 2*B*,*C*) and Y (Fig. 2*D*,*E*), respectively, match the firing plots and confirm the long-range modulation with position at both predictive and feedback τ values (bootstrap statistical threshold, *p* = 0.02^a^ for all *p*-values see Table 1 for statistical details).

**Table 1:** Statistical table

Line	Data structure	Test	Power
a	Non-normal (bootstrapped R^2^)	Statistical threshold (mean + 4 SD)	*p* = 0.02
b	Normal (encoding similarity, behavioral interaction)	Pearson correlation	*p* = 0.002
c	Normal (encoding similarity, behavioral interaction)	Pearson correlation	*p* = 0.003
d	Normal (encoding similarity, behavioral interaction)	Pearson correlation	*p* = 0.01
e	Normal (bootstrapped behavioral noise)	Statistical threshold (mean ± 4 SD)	*p* < 0.0001

Similar long-range modulation in the simple spike discharge occurs with velocity (VX and VY). As shown for another Purkinje cell, simple spike firing increases in the upper right quadrant of the velocity space from -2000 to -1000 ms with a maximum at approximately -1700 ms. Similarly, there is long-term modulation with velocity at feedback lags characterized by increased firing in the lower right quadrant of the workspace that peaks at approximately -800 ms. Both the R^2^ and β temporal profiles reveal significant and strong correlations and modulation in the simple spike firing, respectively, from -2000 to -1000 ms in VY and from -200 to +2000 ms in both VX and VY (bootstrap statistical threshold, *p* = 0.02^a^, Fig. 3*B–E*). There is also short-range modulation of the simple spike firing with VX and VY with peaks at -200 and 300 ms, respectively (bootstrap statistical threshold, *p* = 0.02^a^).

Finally, correlations at extended lead and lag times also occur with performance errors. As shown in Figure 4, plots of the simple spike firing in relation to position errors (XE and YE) and the results of the regression analyses reveal strong predictive modulation beginning at -1600 ms as well as modulation at lags from 800 to 1800 ms. For this Purkinje cell, the strongest modulation is with YE. As these examples illustrate, simple spike firing can both lead and lag motor behavior over a ±2000-ms window.

### Population results during tracking

**Figure 5.**
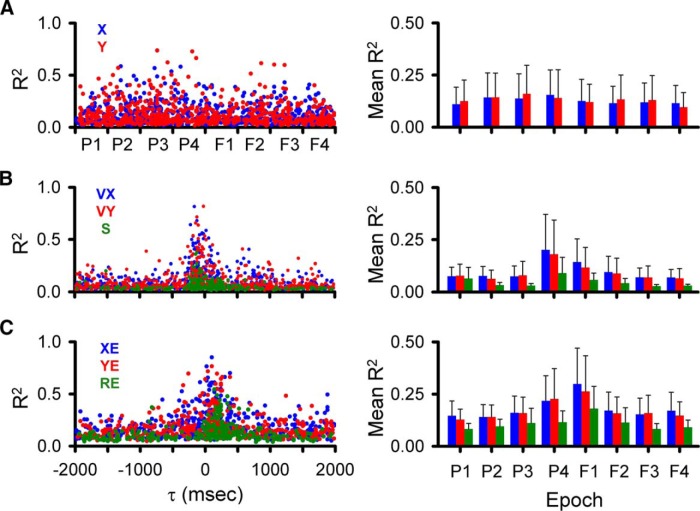
Temporal distribution of simple spike peak and averaged R^2^ values during the track period. ***A***, Distributions of significant R^2^ peaks for position (left panel) and averaged R^2^ (right panel) for each of the predictive and feedback epochs (X, blue; Y, red). ***B***, Similar distributions of significant R^2^ peaks (left panel) and averaged R^2^ (right panel) for velocity (VX, blue; VY, red) and speed (S, green). ***C***, Distributions of significant R^2^ peaks (left panel) and averaged R^2^ (right panel) for position error (XE, blue; YE, red) and radial errors (RE, green). Epochs are P1: -2000 to -1500 ms, P2: -1500 to -1000 ms, P3: -1000 to -500 ms, P4: -500-0 ms, F1: 0-500 ms, F2: 500-1000 ms, F3: 1000-1500 ms, F4: 1500-2000 ms. Same epochs are used in subsequent figures.

As detailed in the Materials and Methods and illustrated in Figures 2–4, we employed a very conservative criterion to define a significant correlation between the simple spike firing and a parameter based on shuffled trials, requiring that the R^2^ exceed the mean + 4 SDs of the results from shuffled trials. Using this definition of significance, we determined for each parameter the magnitude and timing of the largest significant peak R^2^s in each of the predictive and feedback 500-ms epochs (bootstrap statistical threshold, *p* = 0.02^a^; Fig. 5).

While for most parameters, the largest R^2^ values are in the -500- to 500-ms epochs, significant simple spike modulation occurs frequently for the longer epochs (Fig. 5, left column). In the longest feedforward epoch (P1, -1500 to -2000 ms), 152 Purkinje cells (83%) have at least one R^2^ profile with a significant peak among all parameters and an average of 2.3 ± 1.3 significant peaks. For the longest feedback epoch (F4), 159 cells (87%) have at least one significant peak and an average of 2.3 ± 1.2 significant peaks. Similar numbers of Purkinje cells (168 ± 7 cells) have at least one significant peak in any of the four intermediate epochs spanning -1500 to -500 (P2 and P3) and 500-1500 ms (F2 and F3) with an average of 2.5 ± 1.2 significant peaks per cell. For each of the two short-range epochs covering -500-500 ms (P4 and F1), 173 Purkinje cells have at least one significant peak, with an average 3.8 ± 1.6 significant peaks per cell. With the exception of the shortest-range epochs (P4 and F1), the individual components of position (X, Y), velocity (VX, VY), and position error (XE, YE) have more frequent representations (59 ± 20 Purkinje cells on average) than S and RE (19 ± 7 Purkinje cells on average).

Averaging the R^2^ values for each parameter across the 500-ms epochs (Fig. 5, right column) confirms that the weakest representations are with S and RE. The strength of position encoding (X, Y) is relatively constant. For the remaining parameters (VX, VY, S, XE, YE, RE), the average R^2^ values show that while encoding strength is greatest for the shortest-range epochs (P4 and F1), there is a strong encoding in all epochs.

### Control analyses

**Figure 6.**
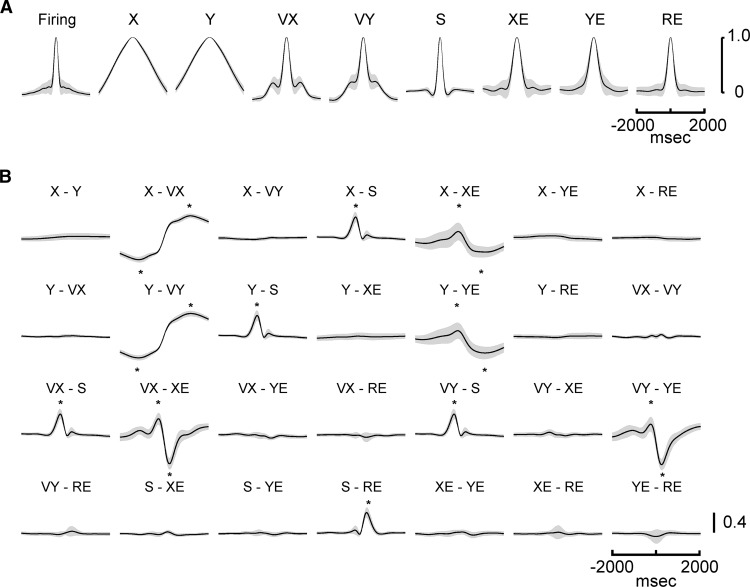
Behavioral parameters temporal structure and interactions. ***A***, Autocorrelograms of the simple spike firing and behavioral parameters. ***B***, Cross-correlograms for all pairs of kinematic and error parameters. An * marks the time of a positive or negative correlation between parameters that exceeds a threshold of *ρ* < -0.1 or *ρ* > 0.1, respectively. Mean (solid line) ± SD (gray area) of autocorrelograms and cross-correlations computed over entire data set from the 183 Purkinje cells.

Because the finding of simple spike modulation that leads and/or lags the kinematics and performance errors over these extended times is novel and potentially controversial, it is imperative to demonstrate that the results are neither spurious nor the result of inherent confounds, such as inter- or intratemporal correlations in either the spike trains or individual behavioral parameters. Therefore, in addition to using the strict criterion for significance, we performed several control analyses. To address the question of temporal correlations within a parameter, the autocorrelations (mean ± SD) were determined for each kinematic and error parameter as well as the simple spike firing of the 183 Purkinje cells (Fig. 6*A*). Most of the autocorrelations have a relatively narrow time span with half-height durations of 440 ms for velocity, 280 ms for speed, 580 ms for position error, 400 ms for radial error, and 280 ms for simple spike firing. Both VX and VY have secondary peaks at ±800 and ±680 ms, respectively. However, the amplitude (ρ value) of these peaks is low (∼0.2), and could account for no more than 4% of the variability. All other local maxima in the autocorrelation profiles are <0.1 and could account for <1% of the variability. In addition, the autocorrelation profiles are highly stereotypic with very small variance across the population of neurons, unlike the widely distributed peak R^2^ times for the regressions of the firing with kinematics or errors (see Figure 5). Furthermore, the autocorrelation of many of the parameters decreases smoothly from the peak at 0 ms, which is quite different from the complex R^2^ temporal profiles of the simple spike firing (Figs. 2–4). Therefore, the temporal structure of the autocorrelations for the behavioral parameters and the simple spike discharge cannot easily explain the firing regression results.

The above arguments are not as straight-forward for position. As expected, the autocorrelations for X and Y extend over longer time courses with a half-width of 2240 ms as position changes slowly. However, there are no secondary peaks in the autocorrelations of X and Y that can explain the multiple peaks and complex temporal structure found in the simple spike regressions (Fig. 5). The regression analysis for a parameter based on using the residuals in the simple spike firing after removing the variability associated with the other parameters was designed to remove correlations among parameters at each τ value. However, this does not necessarily eliminate the correlations among parameters at different τ values. Therefore, we determined the cross-correlations between all possible combinations of parameters (Fig. 6*B*). Although many of the cross-correlations are essentially flat, particularly beyond the ±500-ms epochs, several pairs of parameters have peak correlations (*ρ* < -0.1 or *ρ* > 0.1). However, two observations suggest that these interactions cannot explain the long-range encoding observed in the simple spike firing.

First, the timing of the correlations for all behavioral pairs is very stereotypic, with the minima and maxima at specific leads and lags. In contrast, the times of the R^2^ peaks in the simple spike regression are distributed widely throughout the ±2000-ms window (Fig. 5). Second, we developed a more quantitative assessment of whether the observed short- or long-range correlations between some pairs of behavioral parameters (Fig. 6*B*) contributed to the simple spike encoding of these parameters. As described in the Materials and Methods, for pairs of parameters with a correlation that exceeded a threshold (*ρ* < -0.1 or *ρ* > 0.1), the encoding similarity between the β profiles was determined and then compared with the behavioral interactions across all Purkinje cells. Strong correlations between the encoding similarities and behavioral interactions would demonstrate that the behavioral relationships determine the simple spike encoding. Conversely, weak or nonsignificant correlations would suggest that the simple spike representations of the two parameters, although influenced by the behavioral interactions, also encode independent information.

**Figure 7.**
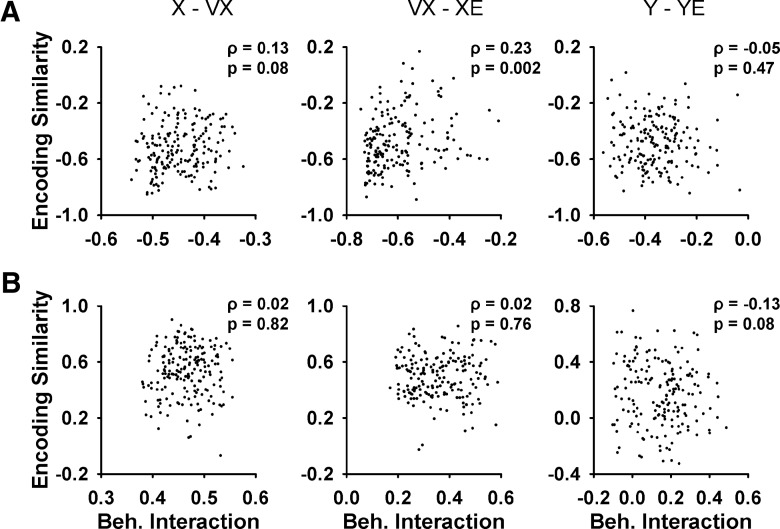
Relation between encoding similarity and behavioral interaction for pairs of parameters with significant correlation. ***A***, Scatter plots of encoding similarity minima versus peak negative behavioral interaction for X and VX, VX and XE, and Y and YE, respectively. ***B***, Scatter plots of encoding similarity maximum versus peak positive behavioral interaction for the same parameters in ***A***. Each scatter plot shows data from all 183 Purkinje cells. The times of the peak negative and positive behavioral interactions for each parameter are shown in Figure 6*B* (*). The Pearson correlation coefficient (*ρ*) and *p* value are included in each scatter plot.

Figure 7 shows examples of this analysis of the relation between encoding similarity and behavioral interaction for three pairs of parameters with the largest correlations. The six scatter plots (three for the maxima and three for the minima of the behavioral interaction) illustrate that encoding similarity is mostly uncorrelated to the behavioral interaction, with the only significant correlation for the minimum of VX with XE (Pearson correlation, *ρ* = 0.23, *p* = 0.002^b^; Fig. 7*A*). For all other pairs, the only additional significant correlations between encoding similarity and behavioral interaction are for the minimum of Y with VY (Pearson correlation, *ρ* = 0.22, *p* = 0.003^c^) and the maximum of S with RE (Pearson correlation, *ρ* = 0.20, *p* = 0.01^d^). Although significant, the magnitudes of these correlations are small. These control analyses show that the temporal correlations among the behavioral parameters do not reliably predict the degree of simple spike encoding throughout the ±2000-ms window and therefore, the simple spike firing provides independent information about behavior at different moments.

### Decoding of kinematics and errors during tracking

**Figure 8.**
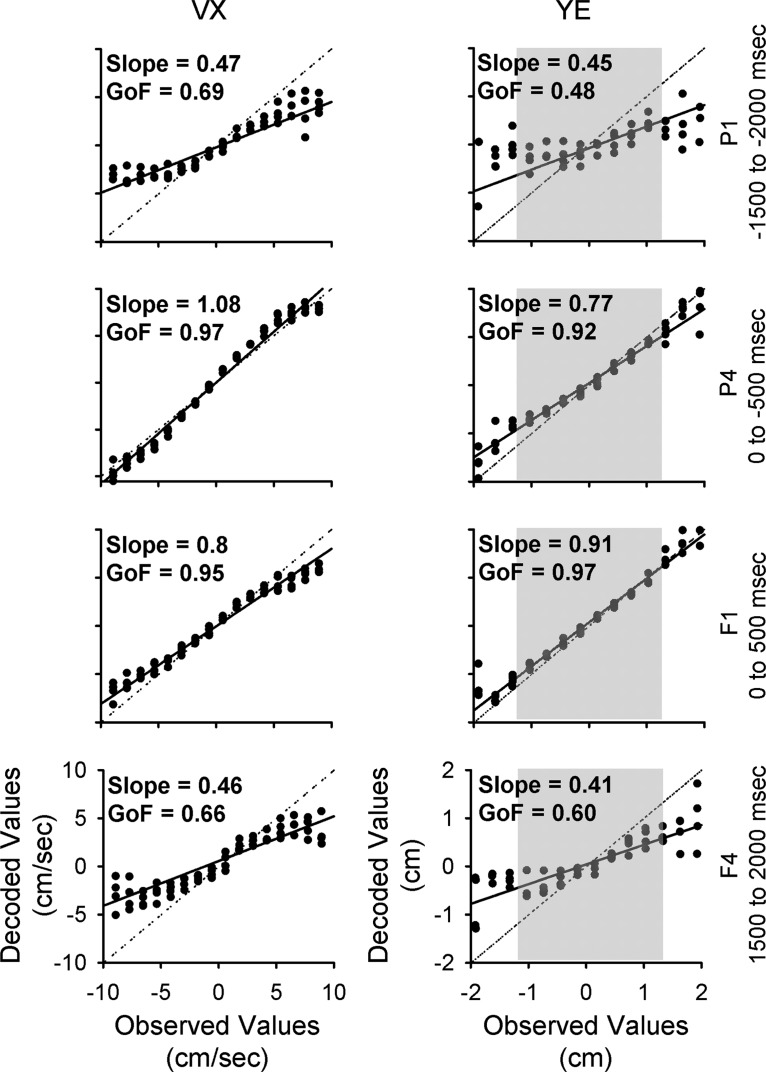
Population decoding of VX and YE in different epochs. Distribution of decoded values versus observed values over 25 decoding repeats. The continuous lines illustrate the slope of each distribution. The dotted lines illustrate a slope of 1, corresponding to perfect decoding. In each row, the population decoding is performed using signals from a specific epoch (indicated on the right of each row). In the right column, the gray shaded region denotes the extent of the target.

A population decoding analysis assessed the quality and accuracy of behavioral information conveyed by the simple spike firing. Figure 8 illustrates several examples of the decoding for VX and YE for the shortest (-500-0 and 0-500 ms, middle panels) and longest (-2000 to -1500 ms, and 1500-2000 ms, top and bottom panels, respectively) epochs. Decoding of VX in the short-range epochs (Fig. 8, middle panels) is excellent with a GoF = 0.97 for the short-range predictive epoch and 0.95 for the short-range feedback epoch. The strong linear correlations between the observed and decoded values with slopes close to 1 (1.08 for predictive and 0.8 for feedback) confirm the excellent decoding and show that the decoded values map most of the velocity working space. For the longest-range epochs (Fig. 8, top and bottom panels), the decoded values increase in variability, particularly near the boundaries of the velocity workspace. At the workspace boundaries, there is also a gradual reduction in the span of decoded to observed values. These two factors decrease the GoF and decoding slope, respectively. For the longest predictive and feedback epochs, the GoFs decrease to 0.69 and 0.66 and decoding slopes to 0.47 and 0.46. The decoding of YE has similar features to VX, with excellent short-range decoding and lower decoding quality at the longest-range epochs.

**Figure 9.**
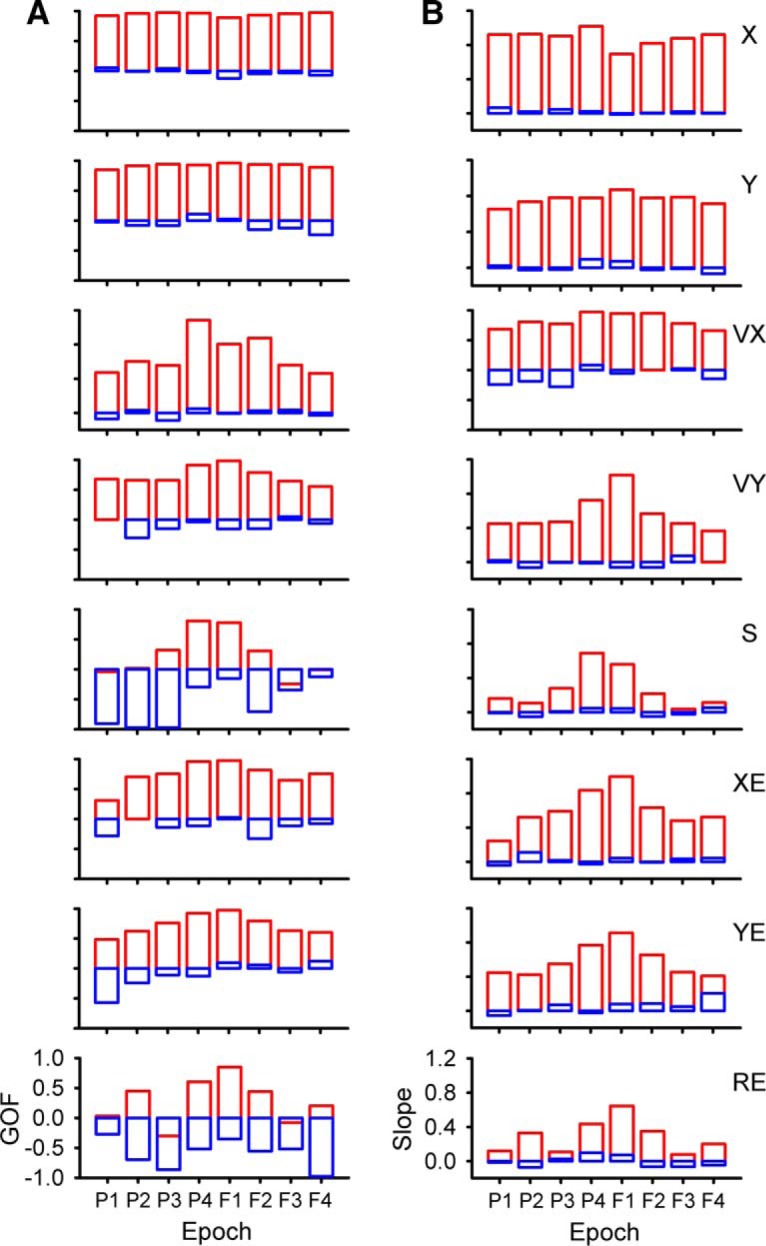
Decoding performance across all epochs during track period. ***A***, GoF for all parameters as identified on column ***B***. ***B***, Decoding slope for all parameters. For both columns, red illustrates population based decoding and blue illustrates chance decoding.

Decoding performance for each behavioral parameter across all epochs is shown in Figure 9. All parameters, without exception, show nearly perfect decoding at the short-range epochs (P4 and F1), which cover the immediate prediction and feedback related to executing current motor commands. The decoding quality measures show that the simple spike firing contains significant information about position, velocity, and position errors over the entire ±2000-ms window.

To better understand the quality of the simple spike decoding, we compared it to random decoding (see Materials and Methods). Random decoding provides little information about the behavior, with GoFs either close to zero or strongly negative and slopes near zero. Although the decoding quality of speed and radial error in the short-range epochs is similar to the other parameters, decoding quality in the long-range epochs is much lower. However, significant information above random is still present in several epochs (-1000 to -500 ms and 500-1000 ms for S and -1500 to -1000 ms and 500-1000 ms for RE). We conclude that the cerebellum encodes rich and simultaneous representations of motor behavior up to 2 s before and after current movement.

### Long-term simple spike modulation with behavior during the hold periods

**Figure 10.**
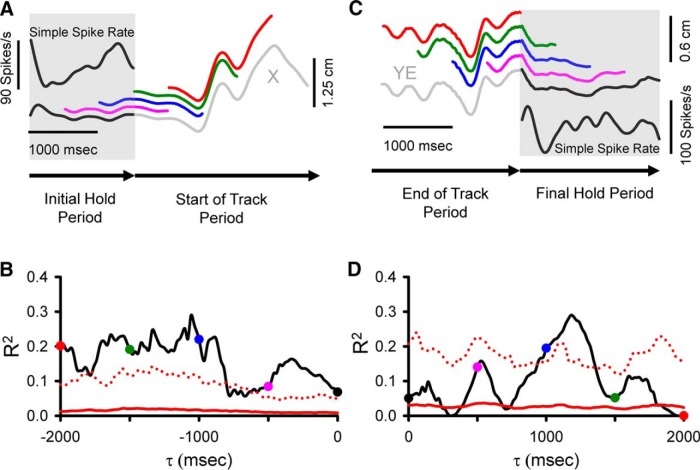
Analyses used to correlate simple spike activity during the hold periods with the behavior parameters. ***A***, Simple spike firing (upper left inset) from a single trial during the initial hold period (gray shadow) is correlated with X-position at different τ values from 0 to -2000 ms using a sliding window of the same width as the initial hold period. Colored traces illustrate X-position at τ = 0 ms (black), τ = -500 ms (pink), τ = -1000 ms (blue), τ = -1500 ms (green), τ = -2000 ms (red). ***B***, For the same cell in A, the R^2^ temporal profile from the regression with X across all trials as a function of τ. ***C***, Simple spike firing (lower right inset) from a single trial during the final hold period (gray shadow) is correlated with position error (YE) recorded in both track (gray segment) and final hold (black segment) periods using a sliding window with the same width as the final hold period moving from 0-2000 ms. Colored segments illustrate the sliding window at τ = 0 ms (black), τ = 500 ms (pink), τ = 1000 ms (blue), τ = 1500 ms (green), τ = 2000 ms (red). ***D***, For the same cell as in ***C***, the R2 temporal profile from the regression with YE encoding across all trials as a function of τ. Arrows at the bottom of ***A*** and ***C*** indicate direction of recording time. ***B***, ***D***, Colored dots coded the same as in ***A*** and ***C***, respectively. Conventions for τ values, and denotation of chance encoding are as in Figure 2.

A second set of analyses evaluated whether the simple spike firing in the initial or final hold periods encodes predictive or feedback behavioral information, respectively. For the initial hold, we tested whether the firing modulates with kinematics or performance errors during the upcoming tracking. The regression analysis compared the simple spike firing during the initial hold to the upcoming position at 20-ms intervals (τ values). The analysis is illustrated in Figure 10*A* showing the actual simple spike firing during the initial hold for a single trial and four example segments of X-position at 500-ms τ values. Note to maintain the same convention as the analyses during the track period, negative τ values denote firing leading the behavior and positive τ values lagging the behavior. Therefore, the 1500-ms initial hold firing (black inset) was regressed to X-position of equal duration, starting at a τ of 0 ms (black segment) to -2000 ms (red segment) in 20-ms steps. As τ increases, the position extends further into the track period. The trial illustrated in Figure 10*A* shows qualitatively that simple spike firing during the initial hold is correlated best with X-position at a τ value of -1500 ms (green X-position trace), which is entirely during the track period. The R^2^ temporal profile across all trials for this example confirms the single trial observation, as the simple spike firing during the initial hold has the largest correlations with upcoming X-position with from -2000 to -1000 ms (bootstrap statistical threshold, *p* = 0.02^a^; Fig. 10*B*).

We also tested whether the firing in the final hold period modulated with kinematics or performance errors during the preceding track period. For the example shown in Figure 10*C*, the simple spike firing during the final hold is regressed to YE at progressive lags, again showing YE at four feedback τ values from 0 to 2000 ms. During this trial, the pattern of simple spike firing during the hold period has the highest correlation with YE at a feedback τ of 1000 ms (blue trace). For this Purkinje cell, the R^2^ plot of the simple spike firing during the final hold period shows significant feedback correlation with YE that peaked at ∼1100 ms (bootstrap statistical threshold, *p* = 0.02^a^; Fig. 10*D*).

**Figure 11.**
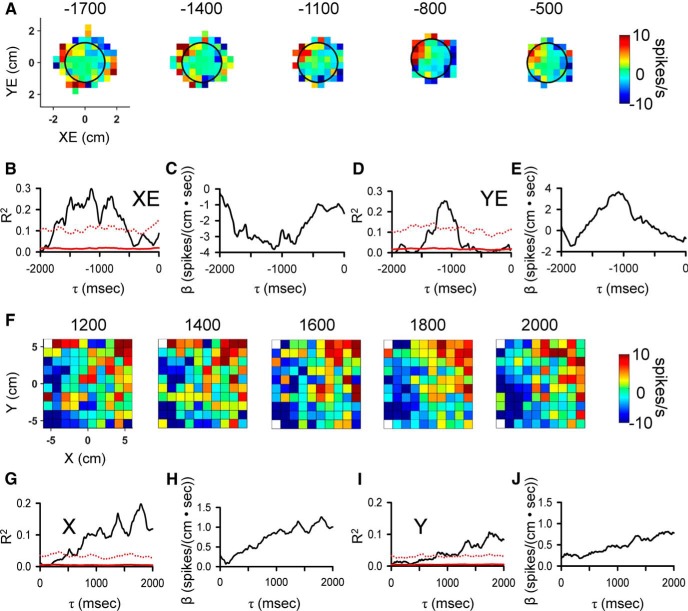
Purkinje cell simple spike modulation during the hold periods in relation to errors and kinematics motor. ***A***, Sequence of firing maps in 300-ms steps of the simple spike modulation in the initial hold with error position. ***B***, ***D***, R^2^ temporal profiles show the strength of XE and YE encoding as function of τ value for this example recording. ***C***, ***E***) β temporal profiles show corresponding simple spike sensitivity during the initial hold to XE and YE as a function of τ value. ***F***, Sequence of firing maps in 200-ms steps of the simple spike modulation during the final hold with hand position for another Purkinje cell. ***G***, ***I***, R^2^ temporal profiles show the strength of X and Y encoding as function of τ value for this neuron. ***H***, ***J***, β temporal profiles show simple spike sensitivity to X and Y as a function of τ value. Color scheme of firing maps, τ values, and denotation of chance encoding are as in Figure 2.

An example of simple spike modulation during the initial hold period in relation to the upcoming position errors is shown in Figure 11*A–*E. The firing plots at several τ values demonstrate strong simple spike modulation that leads XE from -1400 to -500 ms (Fig. 11*A*). The R^2^ and β temporal profiles confirm the presence of significant predictive firing with both XE and YE (bootstrap statistical threshold, *p* = 0.02^a^; Fig. 11*B–E*, respectively), although the stronger and longer modulation occurs with XE. An example of long-range feedback encoding of position during the final hold is shown in Figure 11*F–J*, in which the firing plots exhibit simple spike modulation in the upper right quadrant of the position space at lags of 1200-2000 ms (Fig. 11*F*). The R^2^ and β temporal profiles show the significance, timing and magnitude of the feedback modulation with X and Y (bootstrap statistical threshold, *p* = 0.02^a^; Fig. 11*G–J*, respectively). These examples demonstrate that during the hold periods when the animal is not tracking and relatively stationary, the simple spike activity contains predictive information about the upcoming movement as well as feedback information about the just completed tracking.

**Figure 12.**
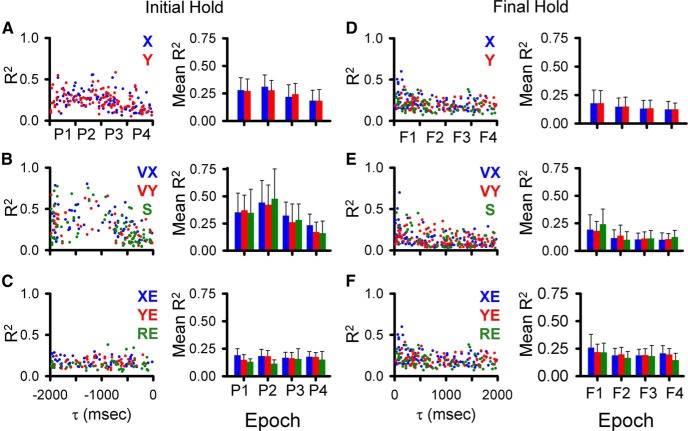
Temporal distribution of peak and averaged R^2^ during the hold periods. ***A–C***, Distribution of significant R^2^ peaks for the initial hold period for each parameter (left panels) and averaged R^2^ (right panels) for the predictive epochs. ***D–F***, Distribution of significant R^2^ peaks for the final hold period for each parameter (left panels), and averaged R^2^ (right panels) in the feedback epochs. Epochs and color-code as in Figure 5.

As done for the analysis of the track period (see Figure 5), the magnitude and timing of the largest significant peak R^2^ was determined for each parameter for the predictive epochs for the initial hold (bootstrap statistical threshold, *p* = 0.02^a^; Fig. 12*A–C*) and for the feedback epochs for the final hold (bootstrap statistical threshold, *p* = 0.02^a^; Fig. 12*D–F*). In the longest feedforward epoch for the initial hold period (-1500 to -2000 ms), 90 Purkinje cells (49%) have a least one R^2^ profile with a significant peak across all parameters and an average of 1.67 ± 1.06 significant peaks. For the longest feedback epoch for the final hold, 108 cells (59%) have a least one significant peak and an average of 1.63 ± 0.86 significant peaks. Similar numbers of Purkinje cells (99 ± 19 cells) have at least one significant peak in any of the four intermediate epochs (P2, P3, F2, F3) with an average of 1.67 ± 0.86 significant peaks per cell. In the shortest-range predictive epoch (P4), the firing in the initial hold period of 95 Purkinje cells has at least one significant peak with an average of 1.5 ± 0.77 peaks. In the shortest-range feedback epoch (F1), the firing in the final hold period of 129 Purkinje cells has at least one significant peak with an average of 1.96 ± 1.16 peaks.

The average R^2^ values for the initial hold period show that position, velocity, and speed are encoded most strongly in the long-range predictive epochs (P1, P2). The error terms have similar R^2^ values for the four predictive epochs. For the final hold period, the strength of the correlation with most parameters is similar across feedback epochs. Therefore, as for simple spike discharge during the track period, the firing in the hold periods exhibits strong correlations with the kinematics and position errors of the upcoming and past tracking over the ±2000-ms time window.

**Figure 13.**
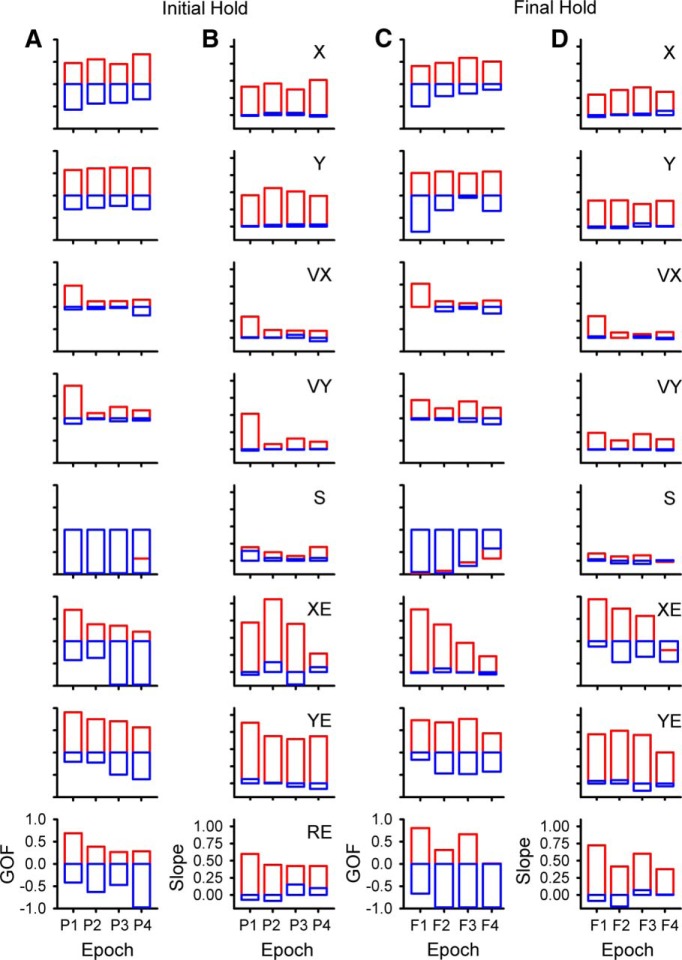
Decoding performance during hold periods. ***A***, ***B***, GoF and decoding slope based on the simple spike firing during the initial hold for each parameter as identified in column ***B***. ***C***, ***D***, GoF and decoding slope based on the simple spike firing during the final hold for each parameter as identified in column ***D***. Epochs as in Figure 5 and color-code as in Figure 9.

Decoding of the simple spike activity in the initial and final hold periods shows that the output of the cerebellar cortex provides surprisingly accurate information about the upcoming motor behavior and of past behavior, respectively, lasting at least 2 s. Based on both GoF and slope, decoding of position (X, Y) during the initial hold is remarkably precise, well above random decoding, and consistent across all four predictive epochs (Fig. 13*A*,*B*, top two panels). This is similar with position decoding observed during the track period (Fig. 9). Position error (XE, YE) and radial error (RE) decoding exceeds random decoding at all predictive epochs with strong decoding for a large lumber of epochs. Velocity decoding based on the simple spike firing in the initial hold is generally less robust, except at the longest-predictive epoch (P1) and speed decoding is at chance level. The strength of decoding based on the firing during the final hold (Fig. 13*C*,*D*) is similar to that observed for the initial hold period. Decoding of position, position error and radial error for the final hold period greatly exceeds random decoding for almost all feedback epochs. Velocity decoding is less strong, except at the shortest feedback epoch (F1) and speed decoding is indistinguishable from chance.

### Little evidence for long-term complex spike modulation with kinematics and performance errors

**Figure 14.**
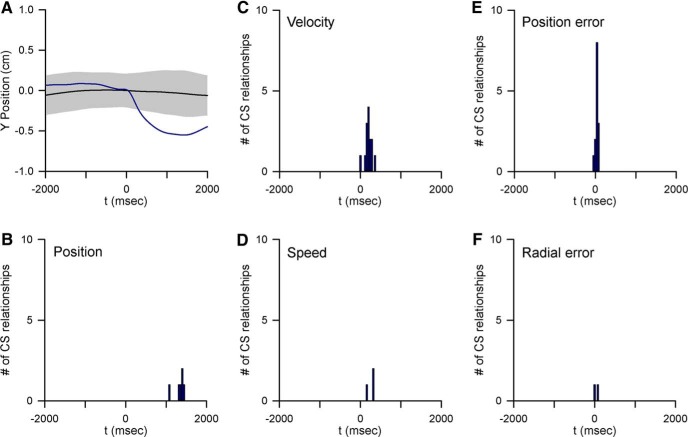
Complex spike-related behavior modulation. ***A***, Example of complex spike-triggered average of Y position (black trace) compared with mean ± 4 SD (gray area) of the noise distribution. ***B***–***F***, Histograms of the temporal relationships between complex spike occurrence and significant behavioral modulation. Note that with the exception of position (***B***), all significant modulations with behavior occur within 500 ms of complex spike discharge. For all plots the data are aligned on complex spike occurrence (t = 0 ms).

In a subset of 40 Purkinje cells, the complex spike discharge was reliably isolated and discriminated throughout the entire recording session. An analysis of both short- and long-term complex spike modulation with each parameter during the track period was undertaken on this group of neurons using complex spike-triggered averaging. In these cells, the average pause in simple spike firing following complex spike discharge was 48.6 ± 85.7 ms with a minimum inactivation period of 10 ms (Streng et al., 2017). With four of the five parameters only short-term complex spike modulation occurred (Fig. 14*B–F*). Only five instances of complex spike long-term modulation with behavior were observed, all with position, as shown for an example Purkinje cell (Fig. 14*A*). For these five long-term complex spike modulations, identified in four cells, the climbing fiber activity preceded a significant change in position (*p* < 0.0001^e^; Fig. 14*B*).

As opposed to this incidental long-range relationship between complex spike discharge and position, the long-range relationship between simple spike activity and behavior in this subpopulation mirrors that found in the entire Purkinje cell population. For position, 80 long-range predictive signals were found in simple spike firing of 32/40 Purkinje cells and 95 long-range feedback signals in 28 cells. For velocity, 60 long-range predictive signals and 64 feedback signals were present in 31 Purkinje cells each. For position error, 29 long-range predictive signals were observed in 23 cells and 44 feedback signals in 26 cells. As in the population, long-range speed and radial error encoding, both predictive and feedback, were less ubiquitous being present in 10 cells each. Therefore, the long-term modulation in the simple spike firing is not associated with or due to similar long-term relationships in the complex spike discharge.

## Discussion

The key finding presented here is that Purkinje cell simple spike firing correlates with kinematics and performance errors over a much longer time horizon than previously reported (Marple-Horvat and Stein, 1987; Fortier et al., 1989; Stone and Lisberger, 1990; Shidara et al., 1993; Fu et al., 1997; Gomi et al., 1998; Coltz et al., 1999; Roitman et al., 2005; Medina and Lisberger, 2009; Dash et al., 2012). Significant modulation commonly occurs at least 2 s before and following many of the behavioral parameters evaluated. Although the strongest modulation occurs between -500 and 500 ms, decoding demonstrates that the information present in the population of Purkinje cells can accurately predict a great deal about the upcoming position, velocity, and position errors and retain signatures of these parameters over this ±2-s period. While speed and radial error exhibit weaker long-range modulation, population decoding during tracking for these two parameters reveals substantial improvement above random decoding. Control analyses show that the long-range encoding is neither spurious nor due to the temporal structure of either the simple spike firing or the individual behavioral parameters. Nor can the simple spike modulation be attributed solely to correlations between parameters. The finding that the simple spike firing in the hold periods encodes similar predictive and feedback information strengthens the view that the cerebellum’s role is not relegated to immediate movement processing but instead operates over a much longer range of time. Finally, analyses of the complex firing in a subset of Purkinje cells reveals little evidence for long-term modulation with behavior, showing that long-term signaling in the simple spike firing is independent of the complex spike discharge.

The presence of long-range predictive information may appear to be somewhat paradoxical in a pseudo-random tracking task. However, to ensure that the task was not prohibitively difficult, the random trajectories were smoothed by low-pass filtering of the target path and implementation of the two-thirds power law for target speed. While the full spatial and temporal course of the trajectories are indeed unpredictable, the smooth target movement allows formation of expectations about upcoming motor behavior, such as the approximate hand or target movement, over 1-2 s. In the -500 to 0-ms epoch, upcoming kinematics and position error can be predicted quite accurately and then decrease with time. At both longer predictive and feedback epochs, the information in the simple spike firing was less able to accurately reconstruct position errors than kinematics. This likely reflects that upcoming position errors are less predictable than kinematics due to increased target movement uncertainty, and that kinematic feedback information benefits from multimodal support, both visual and proprioceptive (Krakauer et al., 1999; Pipereit et al., 2006; Bock and Thomas, 2011), while position error information is visual.

### Limitations of the analyses

It is important to discuss the limitations of the analyses. First, we were not able to determine whether the motor signals extend beyond ±2 s. The pseudo-random tracking trajectories are 8-10 s long. Therefore, to have sufficiently long data, the analysis was restricted to 2 s ahead and following a parameter. However, as shown in the examples (Figs. 2–4), the simple spike modulation is likely to extend somewhat beyond the ±2-s interval. To examine longer time courses of these signals, paradigms with longer trajectories and holding periods would be useful.

Second, while the linear regression analysis based on residual firing completely isolates a parameter at each τ value, the approach does not remove intertemporal correlations. The control analyses demonstrate that long-range correlations among parameters are limited to a few pairs and are very stereotypic (Fig. 7*B*). However, the correlations in the simple spike firing with the parameters are distributed over a much greater range of prediction and feedback times compared with the behavioral correlations (Fig. 5) and encoding similarity did not vary with the behavioral correlation for most pairs of parameters. Therefore, while intertemporal correlations cannot be completely controlled for nor eliminated, these cannot account for prevalence and times of the long-range firing modulations. Irrespective of any behavioral correlations, there is sufficient information in the simple spike firing to decode simultaneously both upcoming and past behavior over the ±2-s window. Finally, while we did not record eye movements, several studies of Purkinje cell discharge in the regions recorded found little evidence for eye movement related activity (Marple-Horvat and Stein, 1990; Mano et al., 1991; Mano et al., 1996; Coltz et al., 1999).

### Functional implications

The long-term simple spike modulation with kinematics and position errors has important implications for understanding the implementation of forward internal models, specifically how performance from past actions informs subsequent actions. Forward models predict the consequences of a motor command and those predictions are compared with the actual sensory feedback to compute sensory prediction errors that are used to update motor commands and guide motor learning (Miall and Wolpert, 1996; Wolpert and Ghahramani, 2000; Mazzoni and Krakauer, 2006; Shadmehr et al., 2010; Wong and Shelhamer, 2011; Taylor and Ivry, 2012; Gaveau et al., 2014). The cerebellum is widely hypothesized to implement forward internal models (Wolpert et al., 1995; Wolpert et al., 1998; Pasalar et al., 2006; Bell et al., 2008; Shadmehr et al., 2010; Popa et al., 2013). The long-range feedback modulation in the simple spike firing provides a mechanism by which the motor system retains information about past performance to both evaluate the consequences of previous motor commands as well as update subsequent commands. Both kinematic and task error information persists over several seconds which suggests that the cerebellum has access to multiple classes of information about past performance in making these computations.

The long-range signals in the simple spike discharge have implications beyond internal models for motor control as these signals provide a neural substrate for aspects of cerebellar function that operate over several seconds. The presence of long-range signals in the hold periods, in the absence of motor commands related to tracking behavior, suggests that cerebellum not only encodes consequences of current motor command but also encodes expectations of future behavior and working memories of past behaviors. As reviewed in the Introduction, lesion and functional imaging studies document cerebellar involvement with motor and nonmotor sequencing and working memory (Doyon et al., 1997; Lu et al., 1998; Nixon and Passingham, 2000; Chen and Desmond, 2005; Molinari et al., 2008; Hautzel et al., 2009; Marvel and Desmond, 2010; Küper et al., 2016). The cerebellum is hypothesized to be part of a network subserving attentional anticipation and working memory that includes the prefrontal and inferior parietal cortices (Posner et al., 1984; Kim et al., 1994; Allen et al., 1997; Knight, 1997; Allen et al., 2005). Event related functional MRI show activation of the cerebellum 2-3 s before the onset of movement and cognitive tasks (Hülsmann et al., 2003). Magnetoencephalography shows that cerebellar activation anticipates sensory stimuli up to 4 s in advance (Tesche and Karhu, 2000). The cerebellum has an active role in this network, as dentate lesions attenuate the readiness potential (i.e., the Bereitschaftspotential; Kitamura et al., 1999), a major anticipatory marker for self-initiated movements generated in the motor cortices (Deecke et al., 1976; Neshige et al., 1988; Ikeda et al., 1995). Another interesting observation is cerebellar involvement in the 3-4 s integration window present in motor, sensory and cognitive processes, considered to represent the “subjective present” (Bechinger et al., 1969; Fraisse, 1984; Mates et al., 1994). In a synchronous finger tapping task, patients with spinocerebellar ataxia types 6 and 31, relatively pure types of cerebellar degeneration, have a shortened temporal integration span compared with the 3 to 4-s span of healthy subjects (Matsuda et al., 2015). It has been postulated that the time window of the subjective present is defined by the cerebellum (Ghajar and Ivry, 2009). Therefore, the long-range signals described here for the motor domain may play a more general role in cerebellar function.

### Source of the long-term simple spike signals

It is unclear whether these long-term signals are synthesized locally or conveyed by afferent input. It has been suggested that the motor error trace observed during reaching is due to synaptic plasticity (Huang and Shadmehr, 2007; Chen-Harris et al., 2008; Ethier et al., 2008; Yang and Lisberger, 2010). There are many forms of short and long-term cerebellar synaptic plasticity that could contribute (for reviews, see Hansel et al., 2001; Ito, 2001; Boyden et al., 2004; Gao et al., 2012). It is also possible that the long-range simple spike modulation is generated locally, to some degree. For both Purkinje cells and molecular layer interneurons, synaptic responses to parallel fiber activation can persist over 20 s (Collin et al., 2009; Wang et al., 2011), providing a mechanism by which information can remain within the cerebellar cortical circuitry.

The present findings suggest another mechanism; signals are held in temporary storage as described for working memory in the cerebral cortex. In one model relevant to these long-range signals in the cerebellum, persistent activity in the frontal and parietal cortices modulates the activity of other brain structures, maintaining specific representations of relevant behavioral attributes (Gazzaley and Nobre, 2012; D’Esposito and Postle, 2015; Nyberg and Eriksson, 2015). The cerebellum has strong closed-loop connections with the cerebral cortex, including the motor, prefrontal and parietal cortices (for reviews, see Schmahmann and Pandya, 1997; Strick et al., 2009; Bostan et al., 2013) and, as reviewed above, forms the networks engaged in attentional anticipation and working memory. Neurons in these cortical regions have feedforward and/or working memory discharge consistent with the time courses shown here for Purkinje cells. For example, motor sequence signals have been described in the prefrontal cortex, supplementary motor area and primary motor cortex (Shima and Tanji, 2000; Lu and Ashe, 2005; Averbeck and Lee, 2007) and preparatory/instructional signals in the supplementary motor area and dorsal premotor cortex (Tanji et al., 1980; Kurata and Wise, 1988; Thach, 2007). Using a similar pseudo-random tracking paradigm, the firing of primary motor cortical neurons also exhibits long-range correlations with kinematics (Paninski et al., 2004). Together these observations suggest a likely source of the long-range signaling in the simple spike firing involves recursive network interactions between the cerebellum and cerebral cortex. Irrespective of the specific mechanisms, the presence of long-range representations of both upcoming and past behavior in Purkinje cell discharge provides a possible neural substrate for movement corrections, anticipatory signals, working memory, and temporal integration across multiple classes of behaviors.

## Synthesis

Reviewing Editor: Trevor Drew, University of Montreal Decisions are customarily a result of the Reviewing Editor and the peer reviewers coming together and discussing their recommendations until a consensus is reached. When revisions are invited, a fact-based synthesis statement explaining their decision and outlining what is needed to prepare a revision will be listed below. The following reviewer(s) agreed to reveal their identity: Suryadeep Dash, Chris I. De Zeeuw.

The authors have come a long way in addressing my concerns. However, I still feel they need to show more details on the relation with the complex spikes. It is very unsatisfactory to refer to another paper in press. How can we and future readers know whether all relevant correlations are in place to make a proper comparison? Even if the main point is show an absence of significant correlations (as the authors suggest for the 2 sec period), the relevant complex spike data should still be shown in this paper for eNeuro.

I think that in the additional figure they should show:

A). Raw traces of single unit PCs with clear CSs, SSs and a clean climbing fiber pause.

B). Histograms of CS activity during the same paradigms as used for the current SS recordings, highlighting the activity during the relevant 2 sec events.

C). Population analyses showing the presence or absence of correlations with CSs during the same paradigms.

In the text they should indicate to what extent all cells had both CSs and SSs and whether the CF pauses were all clean. If some cells did not have a clean CF pause, they should indicate why they concluded that these signals

The authors have simply presented their figure 1 as the visual abstract. This will not be understandable to most of the readers without reading the methods section. If the authors wish to present a visual abstract it should be more informative
